# Dolutegravir twice-daily dosing in children with HIV-associated tuberculosis: a pharmacokinetic and safety study within the open-label, multicentre, randomised, non-inferiority ODYSSEY trial

**DOI:** 10.1016/S2352-3018(22)00160-6

**Published:** 2022-07-19

**Authors:** Anna Turkova, Hylke Waalewijn, Man K Chan, Pauline D J Bollen, Mutsa F Bwakura-Dangarembizi, Adeodata R Kekitiinwa, Mark F Cotton, Abbas Lugemwa, Ebrahim Variava, Grace Miriam Ahimbisibwe, Ussanee Srirompotong, Vivian Mumbiro, Pauline Amuge, Peter Zuidewind, Shabinah Ali, Cissy M Kityo, Moherndran Archary, Rashida A Ferrand, Avy Violari, Diana M Gibb, David M Burger, Deborah Ford, Angela Colbers, ODYSSEY Trial Team, Amina Farhana Mehar (nee Abdulla), Pattamukkil Abraham, Elaine Abrams, Judith Acero, Gerald Muzorah Agaba, Grace Ahimbisibwe, Barbara Ainebyoona, Winnie Akobye, Yasmeen Akhalwaya, Nazim Akoojee, Shabinah S. Ali, Pauline Amuge, Catherine Andrea, Maria Angeles Muñoz Fernandez, Rogers Ankunda, Diana Antonia Rutebarika, Suvaporn Anugulruengkitt, Tsitsi Apollo, Moherndran Archary, Ronelle Arendze, Juliet Ategeka, Eunice Atim, Lorna Atwine, Abdel Babiker, Sarah Babirye, Enock Babu, Edward Bagirigomwa, Angella Baita, David Balamusani, Patsy Baliram, David Baliruno, Colin Ball, Henry Balwa, Alasdair Bamford, Srini Bandi, Dominique Barker, Linda Barlow-Mosha, Dickson Bbuye, Shazia Begum, Osee Behuhuma, Sarah Bernays, Rogers Besigye, Maria Bester, Joyline Bhiri, Davide Bilardi, Kristien Bird, Pauline Bollen, Chiara Borg, Anne-Marie Borges Da Silva, Jackie Brown, Elena Bruno, Torsak Bunupuradah, David Burger, Nomzamo Buthelezi, Mutsa Bwakura-Dangarembizi, Africanus Byaruhanga, Joanna Calvert, Petronelle Casey, Haseena Cassim, Sphiwee Cebekhulu, Sanuphong Chailert, Suwalai Chalermpantmetagul, Wanna Chamjamrat, Man Chan, Precious Chandiwana, Thannapat Chankun, Sararut Chanthaburanun, Nuttawut Chanto, Ennie Chidziva, Minenhle Chikowore, Joy Chimanzi, Dujrudee Chinwong, Stuart Chitongo, Moses Chitsamatanga, Joshua Choga, Duangrat Chutima, Polly Clayden, Alexandra Coelho, Angela Colbers, Alexandra Compagnucci, Ana Constança Mendes, Magda Conway, Mark F. Cotton, Jane Crawley, Tim R. Cressey, Jacky Crisp, Ana Cristina Matos, Sumaya Dadan, Jacqui Daglish, Siva Danaviah, Tseleng Daniel, Anita De Rossi, Sukanda Denjanta, Els Dobbels, Maria Dowie, Prosper Dube, Benedictor Dube, Nimisha Dudakia, Alice Elwana, Cristina Epalza, David Eram, Juan Erasmus, Peter Erim, Luis Escosa Garcia, Zaakirah Essack, Carolina Estepa, Monica Etima, Alexandre Fernandes, Maite Fernandez, Felicity Fitzgerald, Jacquie Flynn, Deborah Ford, Claudia Fortuny Guasch, Caroline Foster, George Fourie, Yolandie Fourie, Sophie Foxall, Derusha Frank, Kate Gandhi, India Garcia, Kathleen Gartner, Joshua Gasa, Gugu Gasa, Carlo Giaquinto, Diana M. Gibb, Coral Gomez Rico, Daniel Gomez-Pena, Secrecy Gondo, Anna Goodman, Maria Gorreti Nakalema, Winnie Gozhora, Pisut Greetanukroh, Biobanco Gregorio Maranon, Tiziana Grossele, Shamiso Gwande, Tapiwa Gwaze, Tsitsi Gwenzi, James Hakim, Emmanuel Hakiza, Abdul Hamid Kaka, Ashley Harley, Mornay Isaacs, Richard Isabirye, Wilber Ishemunyoro, Tom Jacobs, Lungile Jafta, Nasir Jamil, Anita Janse Janse van Rensburg, Vinesh Jeaven, Maria José Mellado Peña, Gonzague Jourdain, Katabalwa Juliet, Thidarat Jumpimai, Raungwit Junkaew, Thidarat Jupimai, Winfred Kaahwa, Mildred Kabasonga, Olivia Kaboggoza, Rose Jacqueline Kadhuba, Ampika Kaewbundit, Kanyanee Kaewmamueng, Bosco Kafufu, Brenda Kakayi, Phakamas Kamboua, Suparat Kanjanavanit, Gladys Kasangaki, Naruporn Kasipong, Miriam Kasozi, Hajira Kataike, Chrispus Katemba, Elizabeth Kaudha, Nkata Kekane, Adeodata R. Kekitiinwa, Edridah Keminyeto, Woottichai Khamduang, Warunee Khamjakkaew, Jiraporn Khamkon, Sasipass Khannak, Orapin Khatngam, Tassawan Khayanchoomnoom, Busi Khumalo, Mirriam Khunene, Suwimon Khusuwan, Phionah Kibalama, Robinah Kibenge, Anthony Kirabira, Cissy M. Kityo, Lameck Kiyimba, Nigel Klein, Soraya Klinprung, Robin Kobbe, Olivia Kobusingye, Josephine Kobusungye, Areerat Kongponoi, Christoph Königs, Olivier Koole, Christelle Kouakam, Nitinart Krueduangkam, Namthip Kruenual, Nuananong Kunjaroenrut, Raymonds Kyambadde, Priscilla Kyobutungi, Flavia Kyomuhendo, Erinah Kyomukama, Reshma Lakha, Cleopatra Langa, Laddawan Laomanit, Emily Lebotsa, Prattana Leenasirimakul, Lawrence Lekku, Sarah Lensen, Valériane Leroy, Jin Li, Afaaf Liberty, Juthamas Limplertjareanwanich, Emma Little, Abbas Lugemwa, Ezra Lutalo, Jose Luis Jimenez, Hermione Lyall, Candice MacDonald, Gladness Machache, Penelope Madlala, Tryphina Madonsela, Nomfundo Maduna, Joel Maena, Apicha Mahanontharit, Collin Makanga, Candice Makola, Shafic Makumbi, Lucille Malgraaf, Angelous Mamiane, Felicia Mantkowski, Wendy Mapfumo, Laura Marques, Agnes Mary Mugagga, Lindiwe Maseko, Tshepiso Masienyane, Ruth Mathiba, Farai Matimba, Sajeeda Mawlana, Emmanuel Mayanja, Fatima Mayat, Ritah Mbabazi, Nokuthula Mbadaliga, Faith Mbasani, Kathleen McClaughlin, Helen McIlleron, Watchara Meethaisong, Patricia Mendez Garcia, Annet Miwanda, Carlota Miranda, Siphiwe Mkhize, Kgosimang Mmolawa, Rosie Mngqibisa, Fatima Mohamed, Tumelo Moloantoa, Maletsatsi Monametsi, Samuel Montero, Cecilia L. Moore, Rejoice Mosia, Columbus Moyo, Mumsy Mthethwa, Shepherd Mudzingwa, Tawona Mudzviti, Hilda Mujuru, Emmanuel Mujyambere, Trust Mukanganiki, Cynthia Mukisa Williams, Mark Mulder, Disan Mulima, Alice Mulindwa, Vivian Mumbiro, Zivai Mupambireyi, Alba Murciano Cabeza, Herbert Murungi, Dorothy Murungu, Sandra Musarurwa, Victor Musiime, Alex V. Musiime, Maria Musisi, Philippa Musoke, Barbara Musoke Nakirya, Godfrey Musoro, Sharif Musumba, Sobia Mustafa, Shirley Mutsai, Phyllis Mwesigwa Rubondo, Mariam Naabalamba, Immaculate Nagawa, Allemah Naidoo, Shamim Nakabuye, Sarah Nakabuye, Sarah Nakalanzi, Justine Nalubwama, Annet Nalugo, Stella Nalusiba, Clementine Namajja, Sylvia Namanda, Paula Namayanja, Esther Nambi, Rachael Kikabi Namuddu, Stella Namukwaya, Florence Namuli, Josephine Namusanje, Rosemary Namwanje, Anusha Nanan-kanjee, Annet Nanduudu, Charity Nankunda, Joanita Nankya Baddokwaya, Maria Nannungi, Winnie Nansamba, Kesdao Nanthapisal, Juliet Nanyonjo, Sathaporn Na-Rajsima, Claire Nasaazi, Helena Nascimento, Eleni Nastouli, Wipaporn Natalie Songtaweesin, Kusum Nathoo, Ian Natuhurira, Rashidah Nazzinda, Thabisa Ncgaba, Milly Ndigendawani, Makhosonke Ndlovu, Georgina Nentsa, Chaiwat Ngampiyaskul, Ntombenhle Ngcobo, Nicole Ngo Giang Huong, Pia Ngwaru, Ruth Nhema, Emily Ninsiima, Gloria Ninsiima, Misheck Nkalo Phiri, Antoni Noguera Julian, Monica Nolan, Thornthun Noppakaorattanamanee, Muzamil Nsibuka Kisekka, Eniola Nsirim, Rashina Nundlal, Rosita Nunes, Lungile Nyantsa, Mandisa Nyati, Sean O'Riordan, Paul Ocitti Labeja, Denis Odoch, Rachel Oguntimehin, Martin Ojok, Geoffrey Onen, Wilma Orange, Pradthana Ounchanum, Benson Ouma, Andreia Padrao, Deborah Pako, Anna Parker, Malgorzata Pasko-Szcech, Reena Patel, Rukchanok Peongjakta, Turian Petpranee, Tasmin Phillips, Jackie Philps, Laura Picault, Sonja Pieterse, Helena Pinheiro, Supawadee Pongprapass, Anton Pozniak, Andrew Prendergast, Luis Prieto Tato, Patcharee Puangmalai, Thanyawee Puthanakit, Modiehi Rakgokong, Helena Ramos, Nastassja Ramsagar, Cornelius Rau, Yoann Riault, Pablo Rojo Conejo, Basiimwa Roy Clark, Eddie Rubanga, Baker Rubinga, Chutima Ruklao, Pattira Runarassamee, Diana Antonia Rutebarika, Chalermpong Saenjum, Chayakorn Saewtrakool, Yacine Saidi, Talia Sainz Costa, Chutima Saisaengjan, Rebecca Sakwa, Tatiana Sarfati, Noshalaza Sbisi, Dihedile Scheppers, Stephan Schultze-Strasser, Ulf Schulze-Sturm, Karen Scott, Janet Seeley, Robert Serunjogi, Leora Sewnarain, Clare Shakeshaft, Subashinie Sidhoo, Mercy Shibemba, Delane Shingadia, Sheleika Singh, Wasna Sirirungsi, Sibongile Sithebe, Theresa Smit, Kurt Smith, Marlize Smuts, Moira Spyer, Worathip Sripaoraya, Ussanee Srirompotong, Warunee Srisuk, Mark Ssenyonga, Patamawadee Sudsaard, Praornsuda Sukrakanchana, Pathanee Tearsansern, Carla Teixeira, Kanchana Than-in-at, Thitiwat Thapwai, Yupawan Thaweesombat, Jutarat Thewsoongnoen, Rodolphe Thiébaut, Margaret Thomason, Laura Thrasyvoulou, Khanungnit Thungkham, Judith Tikabibamu, Gloria Tinago, Ketmookda Trairat, Gareth Tudor-Williams, Mercy Tukamushaba, Deogratiuos Tukwasibwe, Julius Tumusiime, Joana Tuna, Anna Turkova, Rebecca Turner, Arttasid Udomvised, Aasia Vadee, Hesti Van Huyssteen, Nadine Van Looy, Ebrahim Variava, Yvonne Vaughan-Gordon, Giulio Vecchia, Avy Violari, Richard Vowden, Hylke Waalewijn, Rebecca Wampamba, Steve Welch, Ian Weller, Sibusisiwe Weza, Ellen White, Ian White, Kaja Widuch, Helen Wilkes, Sookpanee Wimonklang, Ben Wynne, Pacharaporn Yingyong, Zaam Zinda Nakawungu, Peter Zuidewind

**Affiliations:** a Medical Research Council Clinical Trials Unit, University College London, London, UK; b Department of Pharmacy, Radboud Institute for Health Sciences, Radboud University Medical Center, Nijmegen, Netherlands; c University of Zimbabwe Clinical Research Centre, Harare, Zimbabwe; d Baylor College of Medicine, Kampala, Uganda; e Children's Infectious Diseases Clinical Research Unit, Family Center for Research with Ubuntu, Department of Paediatrics and Child Health, University of Stellenbosch, Cape Town, South Africa; f Joint Clinical Research Centre, Mbarara, Uganda; g Perinatal HIV Research Unit, University of the Witwatersrand, Johannesburg, South Africa; h Makerere University–Johns Hopkins University Research Collaboration, Kampala, Uganda; i Pediatric Department, Khon Kaen Hospital, Khon Kaen, Thailand; j Joint Clinical Research Centre, Kampala, Uganda; k Department of Paediatrics and Child Health, King Edward VIII Hospital, Enhancing Care Foundation, University of KwaZulu-Natal, Durban, South Africa; l London School of Hygiene & Tropical Medicine, London, UK

## Abstract

**Background:**

Children with HIV-associated tuberculosis (TB) have few antiretroviral therapy (ART) options. We aimed to evaluate the safety and pharmacokinetics of dolutegravir twice-daily dosing in children receiving rifampicin for HIV-associated TB.

**Methods:**

We nested a two-period, fixed-order pharmacokinetic substudy within the open-label, multicentre, randomised, controlled, non-inferiority ODYSSEY trial at research centres in South Africa, Uganda, and Zimbabwe. Children (aged 4 weeks to <18 years) with HIV-associated TB who were receiving rifampicin and twice-daily dolutegravir were eligible for inclusion. We did a 12-h pharmacokinetic profile on rifampicin and twice-daily dolutegravir and a 24-h profile on once-daily dolutegravir. Geometric mean ratios for trough plasma concentration (C_trough_), area under the plasma concentration time curve from 0 h to 24 h after dosing (AUC_0–24 h_), and maximum plasma concentration (C_max_) were used to compare dolutegravir concentrations between substudy days. We assessed rifampicin C_max_ on the first substudy day. All children within ODYSSEY with HIV-associated TB who received rifampicin and twice-daily dolutegravir were included in the safety analysis. We described adverse events reported from starting twice-daily dolutegravir to 30 days after returning to once-daily dolutegravir. This trial is registered with ClinicalTrials.gov (NCT02259127), EudraCT (2014–002632-14), and the ISRCTN registry (ISRCTN91737921).

**Findings:**

Between Sept 20, 2016, and June 28, 2021, 37 children with HIV-associated TB (median age 11·9 years [range 0·4–17·6], 19 [51%] were female and 18 [49%] were male, 36 [97%] in Africa and one [3%] in Thailand) received rifampicin with twice-daily dolutegravir and were included in the safety analysis. 20 (54%) of 37 children enrolled in the pharmacokinetic substudy, 14 of whom contributed at least one evaluable pharmacokinetic curve for dolutegravir, including 12 who had within-participant comparisons. Geometric mean ratios for rifampicin and twice-daily dolutegravir versus once-daily dolutegravir were 1·51 (90% CI 1·08–2·11) for C_trough_, 1·23 (0·99–1·53) for AUC_0–24 h_, and 0·94 (0·76–1·16) for C_max_. Individual dolutegravir C_trough_ concentrations were higher than the 90% effective concentration (ie, 0·32 mg/L) in all children receiving rifampicin and twice-daily dolutegravir. Of 18 children with evaluable rifampicin concentrations, 15 (83%) had a C_max_ of less than the optimal target concentration of 8 mg/L. Rifampicin geometric mean C_max_ was 5·1 mg/L (coefficient of variation 71%). During a median follow-up of 31 weeks (IQR 30–40), 15 grade 3 or higher adverse events occurred among 11 (30%) of 37 children, ten serious adverse events occurred among eight (22%) children, including two deaths (one tuberculosis-related death, one death due to traumatic injury); no adverse events, including deaths, were considered related to dolutegravir.

**Interpretation:**

Twice-daily dolutegravir was shown to be safe and sufficient to overcome the rifampicin enzyme-inducing effect in children, and could provide a practical ART option for children with HIV-associated TB.

**Funding:**

Penta Foundation, ViiV Healthcare, UK Medical Research Council.

## Introduction

More than 1 million children develop tuberculosis (TB) worldwide every year and, of these, 50 000 are estimated to have HIV-associated TB.^1^ Children living with HIV are at increased risk of acquiring TB and progressing to active disease with high rates of morbidity and mortality.^2^

Treatment of HIV-associated TB is often complicated by overlapping drug toxicities, immune reconstitution inflammatory syndrome associated with TB (TB-IRIS), and drug–drug interactions between anti-TB and antiretroviral drugs.^3^ Rifampicin is an essential component in the treatment of drug-sensitive TB, but is also a potent inducer of several drug transporters and metabolising enzymes,^4^ causing reduction of plasma concentrations of many antiretroviral drugs. Treatment of children with HIV-associated TB is challenging, as there are few available antiretroviral therapy (ART) options to overcome the enzyme-inducing effect of rifampicin.^3^

**Research in context**

**Evidence before this study**

ODYSSEY, an open-label, multicentre, randomised, controlled, non-inferiority trial, has shown that dolutegravir-based antiretroviral therapy (ART) provides superior efficacy compared with other standard-of-care drugs and is safe for the treatment of children with HIV starting first-line or second-line therapy. Rifampicin, a key component of treatment regimens for drug-susceptible tuberculosis (TB), interacts substantially with dolutegravir, leading to a lower effective dolutegravir dose. There were no data before this study on dolutegravir dose adjustment in children with TB receiving rifampicin-containing anti-TB treatment.

We searched PubMed on April 15, 2022, with no date restrictions specified, for studies of dolutegravir co-administered with rifampicin, using the terms “dolutegravir”[Title/Abstract] AND (“rifampicin”[Title/Abstract] OR “rifampin”[Title/Abstract]). The search yielded two pharmacokinetic drug-interaction phase 1 studies in adult volunteers without HIV or TB, one phase 2 trial in adults living with HIV, one population pharmacokinetic modelling study, and two adult cohort studies.

Overall, adult studies have shown that co-administration of twice-daily dolutegravir with rifampicin results in target dolutegravir exposure in most people. Once-daily dolutegravir co-administered with rifampicin resulted in lower dolutegravir exposure; however, most people in a large retrospective cohort study in Botswana, who received once-daily dolutegravir while they were treated for TB, reached virological suppression. A non-comparative, phase 2, randomised, controlled trial (RADIANT; NCT03851588) is ongoing to evaluate the efficacy of once-daily dolutegravir 50 mg with rifampicin in adults with HIV-associated TB.

Our search yielded no original studies published in children. A systematic review on pharmacokinetics of antiretroviral and TB drugs for children co-infected with HIV and TB cited a conference abstract from the ODYSSEY pharmacokinetic substudy, the full results of which are published in this paper, and highlighted that children with HIV-associated TB have few ART treatment options.

**Added value of this study**

To our knowledge, this was the first study of dolutegravir co-administered with rifampicin in children with HIV-associated TB, aged from 4 weeks to younger than 18 years. This study provides pharmacokinetic and safety data of using twice-daily dolutegravir alongside rifampicin, and data on HIV suppression, TB outcomes, and rifampicin levels. This study showed that twice-daily dolutegravir (with weight-band daily dose doubled) provided adequate dolutegravir exposures. This approach was safe and effective, with no adverse events considered related to dolutegravir.

**Implications of all the available evidence**

Together with the adult data, this study confirms that twice-daily dolutegravir is efficacious and safe for the treatment of HIV alongside rifampicin-containing anti-TB treatment across all ages. The strategy provides an effective, safe, practical, and readily available treatment option for children. Alignment with adult treatment will make the procurement of drugs easier for HIV programmes and reduce inequity in the access to optimal ART in children with HIV-associated TB.

Dolutegravir-based ART is recommended by WHO as the preferred ART for first-line and second-line treatment in adults and children.^5^ Dolutegravir is primarily metabolised by glucuronidation through UGT1A1, and partly (<10%) through oxidation by CYP3A4.^6^ Co-administration of dolutegravir with rifampicin leads to a substantial reduction in dolutegravir plasma concentrations.^7^ In adults with HIV-associated TB, doubling the daily dose of dolutegravir (50 mg film-coated tablets twice daily) has been shown to overcome the rifampicin-inducing effect and to be effective and well tolerated.^8^ Children might have different pharmacokinetics compared with adults because of developmental differences in absorption, distribution, metabolism, and excretion,^9^ and therefore this important drug–drug interaction requires a separate study in children. We aimed to evaluate the safety and pharmacokinetics of dolutegravir twice-daily dosing in children receiving rifampicin for HIV-associated TB.

## Methods

### Study design and participants

The ODYSSEY trial was an open-label, multicentre, randomised, controlled, non-inferiority trial that evaluated the 96-week efficacy and safety of dolutegravir plus two nucleoside reverse transcriptase inhibitors versus standard of care in 792 children living with HIV, aged from 4 weeks to younger than 18 years, starting first-line or second-line ART in Africa, Europe, and Thailand.^10^ ODYSSEY included two randomised cohorts: the main trial enrolled 707 children with bodyweight of 14 kg or greater,^11^ and an additional randomised cohort enrolled 85 children with bodyweight from 3 kg to less than 14 kg.^12^ Most children from Thailand and Africa in the main trial (613 [94%] of 652 children) and in the cohort with bodyweight of less than 14 kg (70 [95%] of 74) continued in ongoing observational follow-up after completion of the randomised phase, with additional consent.

We nested a two-period, fixed-order, pharmacokinetic drug–drug interaction substudy within the ODYSSEY trial at seven research centres in South Africa, Uganda, and Zimbabwe (four centres recruited). Children with HIV-associated TB who were receiving rifampicin and twice-daily dolutegravir in the randomised phase of the trial, with no other concomitant medications known to have interactions with dolutegravir, and with no acute severe malnutrition, severe diarrhoea, vomiting, renal disease, or liver disease, were eligible for inclusion.

All children in ODYSSEY who had TB treated with rifampicin and twice-daily dolutegravir until June 28, 2021 (follow-up censoring date) were included in the safety population. Children or their carers gave written informed consent and assent as appropriate for participation in the ODYSSEY trial and the pharmacokinetic substudy. The trial, its extended follow-up, and the pharmacokinetic substudy were approved by local ethics committees.

### Procedures

Children with HIV-associated TB receiving dolutegravir and rifampicin had dolutegravir administered twice daily (ie, their daily dose was doubled) from the start of rifampicin dosing if they developed TB during the trial or from the start of dolutegravir-based ART if they had TB before or at trial entry. Twice-daily dolutegravir was switched back to once-daily dosing 2 weeks after stopping rifampicin, because rifampicin induction of drug metabolising enzymes has been shown to persist for approximately 2 weeks after its discontinuation.^13^ Dolutegravir was administered according to the weight-band dosing outlined in the protocol, using 5 mg dispersible tablets and 10 mg, 25 mg, and 50 mg film-coated tablets. During the trial, dolutegravir doses in children with bodyweight from 14 kg to less than 40 kg were increased following the results of weight-band pharmacokinetic substudies,^14^, ^15^ and all study sites adopted the new doses (appendix p 5).

In the pharmacokinetic substudy, the first 12-h pharmacokinetic profile (PK day 1) was conducted in children receiving twice-daily dolutegravir in the last month of anti-TB treatment, and the second 24-h pharmacokinetic profile (PK day 2) was done at 4 weeks after rifampicin discontinuation and 2 weeks after returning to once-daily dolutegravir (appendix p 9). Children had to stay on the same dolutegravir dose for PK day 1 and PK day 2. Children received anti-TB treatment as per WHO 2014 paediatric guidelines^16^ and their country guidelines. Children with bodyweight of less than 25 kg received rifampicin 15 mg/kg (range 10–20); children with bodyweight of 25 kg or more received either the paediatric dose or adult-recommended dose of 10 mg/kg (range 8–12), with a maximum recommended rifampicin dose of 600 mg/day. At the time of PK day 1, children were in the continuation phase of TB treatment and received rifampicin and isoniazid with or without pyrazinamide or ethambutol, at the discretion of the treating clinician.

We collected dolutegravir plasma concentrations at 0, 1, 2, 3, 4, 6, and 12 h after intake of study drugs on PK day 1 and at 0, 1, 2, 3, 4, 6, and 24 h on PK day 2, and rifampicin plasma levels at 0, 2, 4, and 6 h on PK day 1. Children with bodyweight of less than 10 kg fasted for at least 2 h before and 1 h after dosing, and children with bodyweight of 10 kg or more fasted for at least 3 h before and 2 h after dosing, with an overnight fast preferred in this group. Food was provided at set times and the food composition and intake were similar at breakfast on both study days for all children. Plasma samples were separated and stored at –80°C until shipping to the laboratory of the Department of Pharmacy at the Radboud University Medical Center (Nijmegen, Netherlands). Dolutegravir plasma concentrations were measured with a validated ultra-performance liquid chromatography tandem mass spectrometry bioanalytical quantification method with a lower limit of quantification of 0·01 mg/L. Total plasma concentrations of rifampicin were analysed using a validated liquid chromatography mass spectrometry assay with a lower limit of quantification of 0·09 mg/L. Both assays are part of an international quality assurance programme to ensure assay validity.

Children included in the safety population contributed safety data from the start of twice-daily dolutegravir dosing until 30 days after returning to once-daily dosing, death, loss to follow-up, or follow-up censoring date, whichever occurred first. Follow-up in the trial included clinical assessments at weeks 4 and 12, and then 12-weekly after randomisation, with blood samples for haematology and biochemistry collected at weeks 4 and 24, and then 24-weekly or more frequently if clinically indicated. We ascertained serious adverse events, Division of AIDS grade 3–4 clinical and laboratory adverse events, and any events resulting in ART modification.^17^ An independent Endpoint Review Committee, masked to randomised treatment allocation, reviewed all reported adverse events. We determined child-specific TB treatment outcomes for the first TB diagnosis (including incident TB and TB ongoing at randomisation) as favourable (completed TB treatment and remained alive with no TB recurrence) or unfavourable (death from any cause, TB treatment not completed, TB recurrence, withdrawal from the trial, or lost to follow-up) over the period of 72 weeks after the start of twice-daily dolutegravir dosing (proxy for TB treatment start). HIV virological outcomes (suppression to <50 copies per mL and <400 copies per mL) were assessed at 24 weeks after starting twice-daily dolutegravir or at the end of TB treatment, whichever occurred later, for the first TB diagnosis.

### Statistical analysis

We initially enrolled children aged 6 years or older who were receiving dolutegravir doses approved for children at the time (appendix p 5) into the pharmacokinetic substudy. We aimed to enrol five children in two age groups (6 to <12 years and 12 to <18 years), to provide evaluable pharmacokinetic profiles on both study days. This would be sufficient for within-participant comparison of dolutegravir plasma concentrations on PK day 1 versus PK day 2, assuming that within-participant variability (coefficient of variation) for area under the plasma concentration time curve from 0 h to 24 h after dosing (AUC_0–24 h_) and maximum plasma concentration (C_max_) was less than 33%, similar to the pharmacokinetic study in adults without HIV or TB.^7^ The ODYSSEY trial subsequently opened enrolment for children younger than 6 years and moved to dosing using WHO-recommended weight-bands. Dolutegravir dosing in children with bodyweight from 14 kg to less than 40 kg was increased during the trial (appendix p 5), on the basis of the results of the ODYSSEY weight-band pharmacokinetic substudies.^14^, ^18^ We therefore present results by dolutegravir dose and formulation.

Pharmacokinetic assessment visits were only done in children who had been dosed appropriately for the previous 3 days, with dolutegravir and rifampicin on PK day 1 and dolutegravir on PK day 2. Dolutegravir pharmacokinetic profiles for PK day 1 were excluded where there were no data on PK day 2 in any child for the same dose and formulation and vice versa.

We excluded pharmacokinetic profiles if dolutegravir baseline predose concentration (at 0 h) was more than 15 times lower than the end-of-dose interval trough plasma concentration (C_trough_; suspected non-adherence). We conducted non-compartmental analysis and calculation of descriptive statistics for pharmacokinetic parameters. The C_max_ and the time to maximum concentration (T_max_) were directly derived from the plasma concentration time curve. Area under the plasma concentration time curve from 0 h to 12 h after dosing (AUC_0–12 h_) and from 0 h to 24 h after dosing (AUC_0–24 h_) were calculated using the linear up-log down trapezoidal rule for dolutegravir. To estimate dolutegravir AUC_0–24 h_ for twice-daily dosing, we doubled the calculated AUC_0–12 h_.^7^ The apparent elimination half-life (T_1/2_) was calculated by 0·693 divided by λz, where λz is the apparent terminal-phase elimination rate constant and is estimated by linear regression using at least the last three datapoints of logarithmically transformed concentration versus time data. We used linear mixed-effect models with dose as the fixed effect and participant as the random effect for estimation of geometric mean ratios and calculation of 90% CIs.^19^ Similarity of dolutegravir exposure was concluded if the 90% CI for dolutegravir AUC_0–24 h_ and C_max_ on PK day 1 versus PK day 2 was within 0·80–1·25.^19^ We compared dolutegravir geometric mean C_trough_, AUC_0–24 h_, and C_max_ with reference values from adults receiving twice-daily and once-daily dolutegravir with established safety data,^20^, ^21^ and summarised the number of children with C_trough_ of less than the 90% effective concentration (EC_90_) of 0·32 mg/L—the dolutegravir concentration at which 90% of the maximal viral load reduction was achieved in a 10-day monotherapy study.^20^

Rifampicin C_max_ values were compared with the historical optimal range of 8–24 mg/L.^22^ Statistical analyses for pharmacokinetic parameters were performed with Phoenix 64 WinNonlin (version 8.1).

Descriptive analysis of baseline characteristics, adverse events, TB treatment outcomes, and virological outcomes were performed using STATA (version 16.1). The non-pharmacokinetic and pharmacokinetic study groups were compared using Fisher's exact test or χ^2^ test, as appropriate, for categorical variables and the non-parametric Wilcoxon rank-sum test for continuous measures. Grade 3 or higher adverse event rates (per 100 person-years) were calculated as the number of events divided by total person-years at risk times 100 (presented with two-sided 95% CI).

This trial is registered with ClinicalTrials.gov (NCT02259127), EudraCT (2014–002632-14), and the ISRCTN registry (ISRCTN91737921).

### Role of the funding source

Paediatric European Network for Treatment of AIDS Foundation and ViiV Healthcare reviewed the manuscript. Employees of UK Medical Research Council were authors of the paper who were involved in the study design, data collection, data analysis, data interpretation, and writing of the report.

## Results

**Figure 1:**
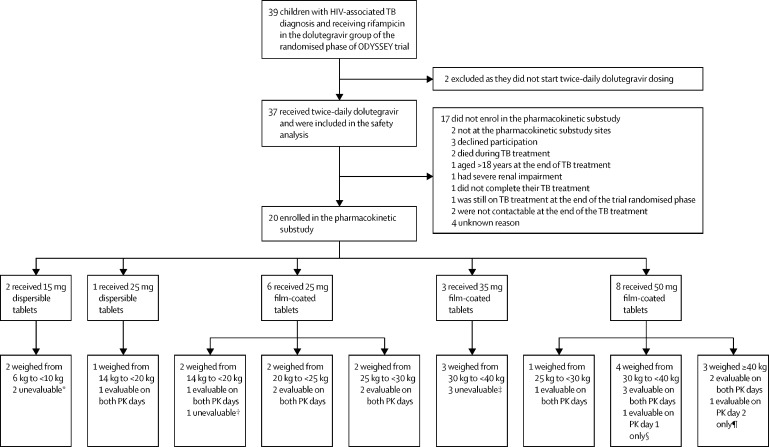
Study profile Dolutegravir doses were protocol-approved doses at the time of pharmacokinetic assessment. Non-adherence to trial medication was predefined as a dolutegravir plasma concentration 24 h after dolutegravir intake (C_trough_) of more than 15 times higher than the baseline concentration. PK=pharmacokinetic. TB=tuberculosis. PK day 1=dolutegravir administered twice daily plus rifampicin. PK day 2=dolutegravir administered once daily. *Both not included in analysis because no data on PK day 1 with rifampicin due to non-adherence for one participant and samples not shipped to laboratory for the other participant; PK day 2 samples not included in linear mixed model analysis because there were no PK day 1 data for this dose and formulation. †PK day 1 unevaluable as the participant took dolutegravir on previous day outside of dosing window and did not return for PK day 2. ‡One participant was non-adherent to trial medication on both PK days; the second participant changed dolutegravir dose from 35 mg film-coated tablet at PK day 1 to 50 mg film-coated tablet on PK day 2 assessment and was non-adherent to trial medication on both PK days; the third participant was non-adherent to trial medication on PK day 1 and did not return for PK day 2. ¶PK day 2 not evaluable due to non-adherence; PK day 1 was evaluable and included in linear mixed model analysis. ||Missing samples on PK day 1 resulting in insufficient number of samples to perform analysis, PK day 2 was evaluable and included in linear mixed model analysis.

Between Sept 20, 2016, and June 28, 2021, 39 children in the dolutegravir group of the ODYSSEY trial were treated for HIV-associated TB with rifampicin-containing treatment; two did not receive a double dolutegravir dose and therefore were excluded from this study (figure 1). Of the 37 children included in this study, 24 had TB at enrolment and 13 developed TB during the trial. The median age at the first use of twice-daily dolutegravir was 11·9 years (range 0·4–17·6), 19 (51%) of 37 were female and 18 (49%) were male, and 36 (97%) were from sub-Saharan Africa (table 1).

**Table 1:** Baseline characteristics at the first use of twice-daily dolutegravir

	**Total safety population (n=37)**	**Not enrolled in pharmacokinetic substudy (n=17)**	**Pharmacokinetic substudy participants (n=20)**	p value*
**ODYSSEY group**				
First-line ART	30 (81%)	13 (76%)	17 (85%)	0·68
Second-line ART	7 (19%)	4 (24%)	3 (15%)	..
**Sex**				
Male	18 (49%)	7 (41%)	11 (55%)	0·40
Female	19 (51%)	10 (59%)	9 (45%)	..
**Country of residence**				
South Africa	9 (24%)	4 (24%)	5 (25%)	0·58
Thailand	1 (3%)	1 (6%)	0 (0%)	..
Uganda	15 (41%)	8 (47%)	7 (35%)	..
Zimbabwe	12 (32%)	4 (24%)	8 (40%)	..
**Ethnic origin**				
Black African	35 (95%)	16 (94%)	19 (95%)	0·72
Asian	1 (3%)	1 (6%)	0 (0%)	..
Mixed Black Indian	1 (3%)	0 (0%)	1 (5%)	..
**Age at dolutegravir dose doubling, years**				
Median (IQR)	11·9 (7·1 to 14·5)	13·6 (8·8 to 16·5)	10·6 (6·8 to 13·0)	0·094
Range	0·4 to 17·6	0·4 to 17·6	0·7 to 15·6	..
<6	5 (14%)	2 (12%)	3 (15%)	..
≥6 to <12	14 (38%)	4 (24%)	10 (50%)	..
≥12 to <18	18 (49%)	11 (65%)	7 (35%)	..
**CD4 percentage**†				
Median (IQR)	14% (5 to 26)	10% (5 to 20)	18% (6 to 30)	0·29
Range	1 to 44%	2 to 33%	1 to 44%	..
<15%	19 (53%)	10 (63%)	9 (45%)	..
≥15%	17 (47%)	6 (38%)	11 (55%)	..
Missing, n	1	1	0	..
**CD4 count, cells per μL**†				
Median (IQR)	262 (109 to 693)	190 (97 to 476)	387 (150 to 814)	0·14
Range	21 to 3538	28 to 1276	21 to 3538	..
<200	14 (39%)	8 (50%)	6 (30%)	..
≥200	22 (61%)	8 (50%)	14 (70%)	..
Missing, n	1	1	0	..
**Log**_10_**viral load, copies per mL**†				
Median (IQR)	3·7 (1·6 to 4·9)	3·7 (1·6 to 4·8)	3·9 (1·7 to 5·0)	0·61
Range	1·3 to 6·6	1·3 to 6·6	1·3 to 5·7	..
<50	12 (32%)	7 (41%)	5 (25%)	..
50–1000	5 (14%)	1 (6%)	4 (20%)	..
≥1000	20 (54%)	9 (53%)	11 (55%)	..
**Type of TB**				
Pulmonary	30 (81%)	14 (82%)	16 (80%)	0·94
Disseminated	3 (8%)	1 (6%)	2 (10%)	..
Abdominal	2 (5%)	1 (6%)	1 (5%)	..
Meningitis	1 (3%)	0 (0%)	1 (5%)	..
Lymph nodes	1 (3%)	1 (6%)	0 (0%)	..
**Timing of first TB diagnosis in trial**				
TB before or at enrolment	24 (65%)	10 (59%)	14 (70%)	0·48
TB after enrolment	13 (35%)	7 (41%)	6 (30%)	..
Median time after enrolment (IQR), weeks	7 (3 to 36)	11 (2 to 98)	6 (4 to 10)	0·67
Range for time after enrolment, weeks	1 to 100	1 to 100	2 to 36	..
**Bodyweight, kg**				
Median (IQR)	26·5 (18·5 to 31·2)	28·7 (23·8 to 41·5)	24·6 (18·3 to 29·3)	0·18
Range	3·5 to 55·2	3·5 to 55·2	6·0 to 40·3	..
**Body-mass index for age, z-score**‡				
Median (IQR)	−1·3 (−2·8 to −0·4)	−1·9 (−3·5 to −0·7)	−1·0 (−1·7 to −0·4)	0·12
Range	−4·5 to 0·8	−4·5 to 0·8	−4·3 to 0·4	..
Less than −3	8 (22%)	6 (35%)	2 (10%)	..
−3 to less than −2	3 (8%)	2 (12%)	1 (5%)	..
−2 to less than 0	19 (51%)	6 (35%)	13 (65%)	..
0 or greater	7 (19%)	3 (18%)	4 (20%)	..

Data are n (%) or median (IQR) unless otherwise stated. ART=antiretroviral therapy. TB=tuberculosis.

* Children not enrolled in the pharmacokinetic substudy and pharmacokinetic substudy participants were compared using Fisher's exact test or χ^2^ test as appropriate for categorical variables, and the non-parametric Wilcoxon rank-sum test for continuous measures.

† Closest measurement within 12 weeks before or after the start of twice-daily dolutegravir.

‡ According to WHO Child Growth Charts and WHO Reference 2007 Charts.

20 (54%) of 37 children enrolled in the pharmacokinetic substudy (figure 1, appendix p 6). The median age was 10·6 years (range 0·7–15·6) and median weight was 24·6 kg (range 6·0–40·3; table 1). Children who participated in the pharmacokinetic substudy were slightly younger and had marginally higher body-mass index and CD4 cell counts than those who did not enrol in the pharmacokinetic substudy; however, the differences were not significant and comparisons were limited by small group numbers (table 1).

**Figure 2:**
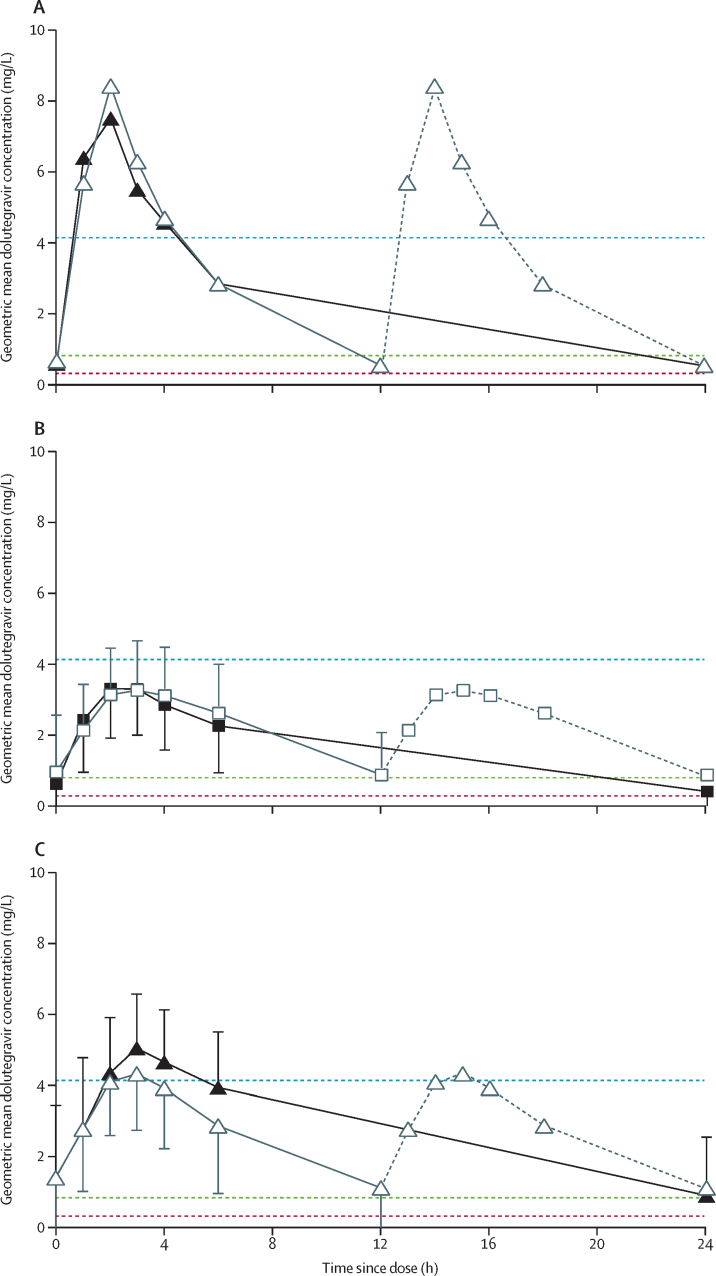
Geometric mean dolutegravir plasma concentration time curves (A) Children receiving dolutegravir 25 mg dispersible tablet once daily (n=1, black solid line) or twice daily with rifampicin (n=1, grey line). (B) Children receiving dolutegravir 25 mg film-coated tablet once daily (n=5, black solid line) or twice daily with rifampicin (n=5, grey line). (C) Children receiving dolutegravir 50 mg film-coated tablet once daily (n=7, black solid line) or twice daily with rifampicin (n=7, grey line). Solid lines in the twice-daily curves indicate observed concentrations; grey dotted lines indicate imputed values by repeating the first 12 h. Geometric mean dolutegravir plasma concentration time curves with adult reference parameters of maximum plasma concentration (C_max_) in adults receiving dolutegravir 50 mg twice daily (blue dotted line), trough plasma concentration (C_trough_) in adults receiving dolutegravir 50 mg once daily (green dotted line), and dolutegravir 90% effective concentration (EC_90_) in the adult dolutegravir 10-day monotherapy study (Min and colleagues, 2011;^20^ red dotted line).

Of 20 children with dolutegravir pharmacokinetic profiles, 12 (60%) had evaluable samples on both PK day 1 and PK day 2 and had within-participant comparisons of pharmacokinetic dolutegravir parameters (figure 2, appendix p 10). Two additional pharmacokinetic profiles were included in the analysis from two children receiving dolutegravir 50 mg film-coated tablets with evaluable samples on only one of the study days (figure 1).

Individual dolutegravir C_trough_ values were higher than 0·32 mg/L (EC_90_) in all children receiving rifampicin with twice-daily dolutegravir (PK day 1), and in all but one participant receiving once-daily dolutegravir (PK day 2) who received the previously licensed dolutegravir dose (table 2, appendix p 11).

**Table 2:** Summary of dolutegravir pharmacokinetic parameters by formulation and dose, and adult reference values

		**Dolutegravir 25 mg dispersible tablet**		**Dolutegravir 25 mg film-coated tablet**		Dolutegravir 50 mg film-coated tablet*		**Adult dolutegravir 50 mg once daily**†**(n=16)**	**Adult dolutegravir 50 mg twice daily**‡**(n=24)**
		Twice daily with rifampicin (n=1)	Once daily (n=1)	Twice daily with rifampicin (n=5)	Once daily (n=5)	Twice daily with rifampicin (n=7)	Once daily (n=7)		
Age at PK days, years		6·2	6·4	8·9 (7·0–10·1)	9·0 (7·2–10·2)	13·0 (12·3–15·9)	13·4 (12·4–16·1)	..	..
Bodyweight, kg		14·6	14·2	24·9 (20·5–27·3)	24·3 (20·0–27·7)	32·0 (31·3–44·3)	33·5 (31·3–47·9)	..	..
Dolutegravir daily dose, mg/kg		3·4	1·8	2·0 (1·8–2·4)	1·0 (0·9–1·3)	3·1 (2·3–3·2)	1·5 (1·0–1·6)	..	..
Rifampicin daily dose, mg/kg		15·4	..	12·0 (11·0–14·6)	..	14·1 (9·6–16·0)	..	..	..
Time between PK day 1 and PK day 2, days§		..	44	..	56 (43–58)	..	57 (51–65)	..	..
Dolutegravir pharmacokinetic parameters									
	C_trough_, mg/L	0·55	0·54	0·90 (16%)	0·43 (58%)	1·11 (99%)	0·89 (53%)	0·83 (26%)	2·12 (47%)
	AUC_0–24 h_, h × mg/L	78·0	54·2	53·4 (21%)	36·2 (29%)	64·4 (61%)	61·9 (43%)	43·4 (20%)	75·1 (35%)
	C_max_, mg/L	8·44	7·52	3·62 (24%)	3·56 (25%)	4·50 (47%)	5·21 (47%)	3·34 (16%)	4·15 (29%)
	T_max_, h	2·00	2·02	3·00 (1·58–4·50)	2·00 (1·50–2·50)	3·00 (2·00–3·00)	3·00 (3·00–3·08)	..	..

Data are median (IQR) for age, weight, drug doses, time between PK day 1 and PK day 2, and T_max_, and geometric means (coefficient of variation) for pharmacokinetic parameters, unless otherwise stated. C_trough_=trough plasma concentration. AUC_0–24 h_=area under the plasma concentration time curve from 0 h to 24 h after dosing. C_max_=maximum plasma concentration. T_max_=time to maximum plasma concentration.

* A total of eight participants had evaluable pharmacokinetics on 50 mg dolutegravir; one participant had an evaluable pharmacokinetic curve on 50 mg dolutegravir twice daily with rifampicin only and one participant on 50 mg dolutegravir once daily only.

† Min and colleagues, 2011.^20^

‡ US Department of Health and Human Services, Food and Drug Administration; highlights of prescribing information, dolutegravir (Tivicay); revised July, 2021.^21^

§ Time between PK day 1 and PK day 2 was calculated for children with both PK day 1 and PK day 2 evaluable curves.

Geometric mean ratios comparing rifampicin and twice-daily dolutegravir versus once-daily dolutegravir for all doses combined were 1·51 (90% CI 1·08–2·11) for C_trough_, 1·23 (0·99–1·53) for AUC_0–24 h_, and 0·94 (0·76–1·16) for C_max_ (table 3). The upper 90% CI bound of the geometric mean ratio for AUC_0–24 h_ and lower 90% CI bound for C_max_were outside the predefined range of 0·80–1·25. As expected, children receiving twice-daily dolutegravir had higher dolutegravir clearance (geometric mean ratio 1·63, 90% CI 1·30–2·04) and lower elimination half-life (0·59, 0·49–0·70) than those receiving once-daily dolutegravir (table 3). In the within-participant comparisons, children did not have consistently higher or lower dolutegravir levels when receiving twice-daily dolutegravir with rifampicin than when receiving once-daily dolutegravir (appendix p 10).

**Table 3:** Geometric mean ratios of pharmacokinetic parameters for twice-daily dolutegravir with rifampicin versus once-daily dolutegravir, by dolutegravir formulation and dose

	**Dolutegravir 25 mg dispersible tablet (n=1)**	**Dolutegravir 25 mg film-coated tablet (n=5)**	**Dolutegravir 50 mg film-coated tablet (n=8)**	**Total (n=14)**
C_trough_, mg/L	1·03	2·10 (1·20–3·67)	1·25 (0·71–2·18)	1·51 (1·08–2·11)
AUC_0–24 h_, h × mg/L*	1·44	1·47 (0·99–2·19)	1·05 (0·74–1·49)	1·23 (0·99–1·53)
C_max_, mg/L	1·12	1·02 (0·73–1·41)	0·86 (0·59–1·27)	0·94 (0·76–1·16)
T_1/2_, h	0·39	0·60 (0·46–0·78)	0·60 (0·44–0·83)	0·59 (0·49–0·70)
Vd/F, L	0·54	0·81 (0·48–1·37)	1·19 (0·81–1·74)	0·96 (0·72–1·28)
CL/F, L/h	1·39	1·36 (0·92–2·01)	1·91 (1·35–2·72)	1·63 (1·30–2·04)

Data are geometric mean ratios (90% CI) of pharmacokinetic parameters for twice-daily dolutegravir administered with rifampicin versus once-daily dolutegravir (reference). C_trough_=trough plasma concentration. AUC_0–24 h_=area under the plasma concentration time curve from 0 h to 24 h after dosing. C_max_=maximum plasma concentration. T_1/2_=apparent elimination half-life. Vd/F=volume of distribution. CL/F=oral clearance. AUC_0–12 h_=area under the plasma concentration time curve from 0 h to 12 h after dosing.

* Individual AUC_0–12 h_ while on twice-daily dolutegravir were multiplied by 2 for extrapolation to AUC_0–24 h_ and used for calculation of geometric mean ratio for AUC_0–24 h_ on twice-daily dolutegravir with rifampicin versus once-daily dolutegravir.

Rifampicin geometric mean C_max_ was 5·1 mg/L (coefficient of variation 71%). Of 18 children with evaluable rifampicin concentrations, 15 (83%) had a C_max_ of less than the optimal target of 8 mg/L (11 (61%) with C_max_ from 4 to <8 mg/L and four (22%) with <4 mg/L (appendix p 7).

Of 37 children included in the safety analysis, 33 had one period of twice-daily dolutegravir co-administered with rifampicin, three children had two periods, and one had three periods. During a median follow-up of 31 weeks (IQR 30–40), 15 reportable grade 3 or higher adverse events occurred among 11 (30%) of 37 children. Ten serious adverse events occurred among eight (22%) children, including two deaths (table 4, appendix p 8). One child discontinued dolutegravir due to abnormal liver function tests and was subsequently diagnosed with hepatitis A virus. No other events resulted in modification of ART. No adverse events or deaths were considered related to dolutegravir by the Endpoint Review Committee. In total, 14·9 person-years of follow-up safety data (58%) were on currently licensed doses (8·4 person-years in 17 children with bodyweight <40 kg and 6·5 person-years in 11 children with bodyweight ≥40 kg); these doses were higher than the initial trial doses in participants with bodyweight from 14 kg to less than 40 kg (appendix p 5). There was no evidence that increased doses of dolutegravir led to higher risks of adverse events (appendix p 8).

**Table 4:** Adverse events

			**Not enrolled in pharmacokinetic substudy (n=17)**	**Pharmacokinetic substudy participants (n=20)**	**Total safety population (n=37)**
Safety follow-up, weeks*					
	Median (IQR)		31 (28–34)	37 (30–43)	31 (30–40)
	Range		8–44	26–147	8–147
Number of safety follow-up periods†					
	1		15 (88%)	18 (90%)	33 (89%)
	2		2 (12%)	1 (5%)	3 (8%)
	3		0 (0%)	1 (5%)	1 (3%)
Number of serious adverse events (number of participants)‡			5 (4)	5 (4)	10 (8)
	Cardiovascular: deep vein thrombosis		0 (0)	1 (1)	1 (1)
	Hepatic: drug-induced liver injury§		1 (1)	0 (0)	1 (1)
	Nervous system: epilepsy, fits, and convulsions		0 (0)	1 (1)	1 (1)
	Injury: trauma¶		1 (1)	0 (0)	1 (1)
	Renal: renal failure chronic		1 (1)	0 (0)	1 (1)
	Skin: rash maculopapular and upper respiratory infection‖		0 (0)	1 (1)	1 (1)
	Systemic: kwashiorkor		1 (1)	0 (0)	1 (1)
	Infectious disease		1 (1)	2 (2)	3 (3)
		Acute febrile episode, undiagnosed	0 (0)	1 (1)	1 (1)
		TB, disseminated or miliary	1 (1)††	1 (1)	2 (2)
Number of grade ≥3 adverse events (number of participants)**			6 (5)	9 (6)	15 (11)
	Cardiovascular: deep vein thrombosis		0 (0)	1 (1)	1 (1)
	Hepatic: drug-induced liver injury§		1 (1)	0 (0)	1 (1)
	Nervous system: epilepsy, fits, and convulsions		0 (0)	1 (1)	1 (1)
	Injury: trauma¶		1 (1)	0 (0)	1 (1)
	Renal: renal failure chronic		1 (1)	0 (0)	1 (1)
	Systemic: kwashiorkor		1 (1)	0 (0)	1 (1)
	Haematological		1 (1)	3 (3)	4 (4)
		Anaemia with clinical symptoms	0 (0)	1 (1)	1 (1)
		Anaemia with no clinical symptoms	0 (0)	1 (1)	1 (1)
		Neutropenia	1 (1)	1 (1)	2 (2)
	Infectious disease		1 (1)	4 (4)	5 (5)
		Acute febrile episode, undiagnosed	0 (0)	1 (1)	1 (1)
		Hepatitis A virus	0 (0)	1 (1)	1 (1)
		TB, disseminated or miliary	1 (1)††	2 (2)	3 (3)
Person-years of follow-up‡‡			9·1	16·4	25·5
Grade ≥3 adverse event rate per 100 person-years (95% CI)			66 (24–143)	55 (25–104)	59 (33–97)
Incidence rate ratio (95% CI) [p value]			1 (ref)	0·83 (0·26–2·84) [0·72]	..
Person-years of follow-up on currently licensed dolutegravir doses			6·3	8·7	14·9
Grade ≥3 adverse event rate per 100 person-years on currently licensed dolutegravir doses (95% CI)			48 (10–140)	46 (13–118)	47 (19–97)

Data are n (n) or n (%) unless otherwise stated. All adverse events except for one were reported in the first safety follow-up period. ART=antiretroviral therapy. TB=tuberculosis.

* Follow-up time was between doubling of dolutegravir dose and 30 days after returning to once-daily dolutegravir or last follow-up visit if not returned to once-daily dolutegravir; two participants did not return to dolutegravir single dose due to death.

† Safety follow-up period was from starting twice-daily dolutegravir with rifampicin to 30 days after returning to once-daily dolutegravir.

‡ Serious adverse events were analysed as episodes, with all components of the same clinical serious adverse event presented as one episode; disease category of the main component is described for serious adverse events composed of multiple components.

§ Hepatitis A; ART-modifying event (dolutegravir was stopped; this event was considered by the Endpoint Review Committee to be unlikely related or unrelated to dolutegravir).

¶ Death due to traumatic accident.

‖ Rash maculopapular (grade 2) and upper respiratory infection (grade 2) were components of the same clinical serious adverse event.

** For grade 3 or worse clinical and laboratory adverse events, each component of the same episode was analysed as a separate event.

†† Serious adverse event reported to have resulted in death in the participant who had a preceding serious adverse event adjudicated as kwashiorkor.

‡‡ Follow-up on lower dolutegravir doses was 10·1 person-years (eight grade ≥3 adverse events in seven children; 79·5 events per 100 person-years) and on doses that were not per protocol or off dolutegravir was 0·5 person-years (no adverse events).

The Endpoint Review Committe considered 11 (30%) of 37 participants to have TB-IRIS, including three diagnoses of paradoxical TB-IRIS among the 24 children who had TB at trial entry and eight diagnoses of unmasking TB-IRIS among 13 children who developed TB during the trial. 32 (86%) of 37 children had favourable TB outcomes, one (3%) had TB recurrence, two (5%) died during TB treatment (one child with kwashiorkor died from disseminated TB [aged 6·8 years] and one infant with pulmonary TB died from accidental injury [aged 0·7 years]), one (3%) completed TB treatment but died due to renal failure (outside safety analysis period for twice-daily dolutegravir and 41 weeks after return to once-daily dosing), and one (3%) completed TB treatment but was subsequently lost to follow-up.

Of 35 children alive at 24 weeks of follow-up, 26 (74%) showed virological suppression to less than 50 copies per mL, and 34 (97%) to less than 400 copies per mL, at 24 weeks after doubling of dolutegravir or at the end of TB treatment for the first TB event, whichever occurred later. Of 12 children who had virological suppression (<50 copies per mL) at the start of their TB treatment, one had low-level viraemia with two viral loads between 50 and 400 copies per mL at the end of TB treatment, and none lost virological control according to the definition of two consecutive viral loads with more than 400 copies per mL.

## Discussion

To our knowledge, this study provides the first pharmacokinetic and safety data for children with HIV-associated TB receiving dolutegravir-based ART and rifampicin.

We confirmed that twice-daily dolutegravir co-administered with rifampicin provided adequate dolutegravir C_trough_ in children and was well tolerated. All children who received rifampicin-containing TB treatment and twice-daily dolutegravir were included in the safety population. Although the pharmacokinetic substudy participants might on average have been slightly healthier than children who did not enrol in the substudy, for the safety analysis it was important not to exclude any children so that all adverse events and deaths were captured.

Rifampicin remains the cornerstone drug for treating drug-sensitive TB, and its drug interactions with antiretrovirals are mitigated by ART modifications. Dolutegravir-based ART is the preferred option in first-line and second-line treatment of HIV,^5^ and in children with HIV-associated TB. Other currently available third-line agents are suboptimal for various reasons.^3^ Efavirenz can be only used for children aged older than 3 years due to its high variability of blood drug concentrations in younger children, and it is not recommended in the context of increasing resistance to non-nucleoside reverse transcriptase inhibitors.^5^ Ritonavir-boosted lopinavir, when co-administered with rifampicin, requires super-boosting with single-entity ritonavir, which is rarely available and often poorly tolerated. Other protease inhibitors and nevirapine are contraindicated with rifampicin. Double-dose raltegravir can be used with rifampicin; however, it has a low barrier to resistance, and treatment failure on raltegravir can compromise future treatment options with later-generation integrase inhibitors. Dolutegravir, however, has superior efficacy and similar safety compared with non-dolutegravir standard-of-care regimens used in children.^11^, ^12^ Dolutegravir is available in dispersible form for young children and is being rolled out for children aged 4 weeks or older globally.^23^ Continuation of dolutegravir with twice-daily dosing while children are receiving rifampicin for TB allows harmonisation of preferred ART regimens across all ages.

Our previously published weight-band pharmacokinetic studies showed that the updated licensed dolutegravir doses for children provide optimal pharmacokinetic profiles.^14^, ^15^ In this study, all children on twice-daily dolutegravir with rifampicin had dolutegravir C_trough_ of greater than the EC_90_ of 0·32 mg/L, which is often used as the minimal individual C_trough_ target associated with effective virological response.^20^, ^24^ Dolutegravir AUC_0–24 h_ with twice-daily dolutegravir and rifampicin was 23% higher than with once-daily dolutegravir without rifampicin, which was similar to the 33% increase observed in adults without HIV or TB.^7^ The geometric mean ratio upper 90% CI bound for AUC_0–24 hv_ and the lower 90% CI bound C_max_ were outside the predefined range of 0·80–1·25, which might have been due to higher variability for dolutegravir AUC_0–24 h_ and C_max_ in children than in adults,^14^, ^15^ and high variability in rifampicin induction.^25^ Given the wide therapeutic window for dolutegravir, a wider target exposure range might be more appropriate for evaluating clinically significant differences between dolutegravir pharmacokinetic profiles on twice-daily dolutegravir and rifampicin compared with once-daily dolutegravir in children.^26^ Although some children had higher AUC_0–24 h_ and C_max_ than the adult reference values, the safety data in this study were reassuring, with no adverse events considered related to dolutegravir.

In line with a paediatric rifampicin pharmacokinetic study,^27^ we found that children who received WHO-recommended rifampicin doses had lower rifampicin C_max_ than adults.^22^ Although most children were successfully treated for TB, rifampicin dosing should be optimised to improve outcomes across all TB disease. If rifampicin dosing is increased in children, this could increase its enzyme-inducing effect, causing a decrease in dolutegravir concentrations. In adults, a three-times (ie, 300%) dose increase of rifampicin caused a 48% decrease in dolutegravir geometric mean C_trough_, with a higher number of people showing C_trough_ of less than 0·32 mg/L (the EC_90_); however, there was no significant difference in the number of people with C_trough_ of less than 0·064 mg/L (the 90% inhibitory concentration or IC_90_) and there were no cases of virological failure among those with low C_trough_.^28^ It is not clear whether this reduction in dolutegravir concentrations is clinically significant, as there is ongoing debate around the dolutegravir minimal C_trough_ target and whether C_trough_ of higher than 0·064 mg/L is sufficient to achieve and maintain virological suppression. Most children in our study received previously licensed dolutegravir doses, which were subsequently increased. This is reassuring, as dolutegravir trough concentrations on the currently approved doses are higher than with previous doses and could potentially withstand an increase in enzyme induction from higher rifampicin dosing. A study with high-dose rifampicin for children on dolutegravir-based ART is ongoing (NCT05069688).

There is some controversy regarding the data on the association between integrase inhibitors and increased risk of TB-IRIS.^29^ Although the numbers are small, numerically more children were adjudicated to have TB-IRIS in the dolutegravir group (11 [2·8%] of 392) than in the standard-of-care group (two [0·5%] of 400) during the ODYSSEY randomised phase.^11^, ^12^ This difference could be related to faster virological suppression on dolutegravir and modestly higher gains in CD4 cell counts observed in ODYSSEY.^11^, ^12^ Of 11 children with TB-IRIS in this study, eight (72%) developed an unmasking form of TB-IRIS (ie, developed a newly diagnosed TB after ART initiation). This form of TB can be prevented by universal TB preventive treatment given to all children with HIV who have initiated ART.^30^ Reassuringly, all children with TB-IRIS, except for one who had severe malnutrition and TB at enrolment, were successfully treated.^11^, ^12^

Virological outcomes were excellent in this study, with 97% of participants showing virological suppression to less than 400 copies per mL at the end of TB treatment or at 24 weeks after ART initiation, whichever occurred later. This finding is similar to the results in the dolutegravir group of the main ODYSSEY trial, where 88% of participants showed virological suppression to less than 400 copies per mL at 24 weeks, 89% at 48 weeks, and 89% at 96 weeks.^11^

A limitation of this study was the small numbers of young children on dispersible tablets and children on film-coated tablets in the weight bands (20 to <40 kg) for which licensed dolutegravir doses were recently increased. However, 17 children and more than 8 person-years of follow-up were included in these groups, providing information on the safety of this approach. Alongside the adult data,^7^, ^8^ this study provides evidence for the efficacy and safety of twice-daily dolutegravir in children receiving rifampicin for HIV-associated TB.

In conclusion, the pharmacokinetic and safety data presented in this study show that a dolutegravir twice-daily dosing strategy can overcome a rifampicin drug interaction and can be given safely to children, providing a practical, effective, and readily available treatment option for children with HIV-associated TB.

## Data sharing

The ODYSSEY data are held at Medical Research Council Clinical Trials Unit at University College London, which encourages optimal use of data by employing a controlled access approach to data sharing, incorporating a transparent and robust system to review requests and provide secure data access consistent with the relevant ethics committee approvals. We will consider all requests for data sharing, which can be initiated by contacting mrcctu.ctuenquiries@ucl.ac.uk.

## Declaration of interests

AT, DF, DMG, and MKC received support from core funding to the Medical Research Council Clinical Trials Unit (grant numbers MCUU_00004/03 and MCUU_00004/07). AC received grants for pregnancy research from Gilead Sciences, ViiV Healthcare, and Merck Group. AT received funding for serving on ViiV Healthcare advisory board. AV served on a Data Safety Monitoring Board for Janssen Pharmaceuticals. DMB received grants from ViiV Healthcare, Gilead Sciences, and Merck Group for the PANNA network; served on the advisory board for Merck Group; participated in the work of the Data Safety Monitoring Board for Janssen Pharmaceuticals; and received funding from ViiV Healthcare and Pfizer for lectures. RAF received Senior fellowship in Clinical Science from Wellcome Trust. AT, AC, DMB, DMG, MA, and PA are members of the WHO-led Paediatric Antiretroviral Working Group (PAWG) and Paediatric Drug Optimisation Group (PADO). AT and PA are members of the WHO-led Child and Adolescent TB Technical Working Group. MA and AC served as PAWG Co-Chairs. MA served as a Vice Chair of the Treatment Scientific Committee of the International Maternal Pediatric Adolescent AIDS Clinical Trials Group (IMPAACT). All other authors declare no competing interests.
