# Supplementary material for: Dolutegravir twice-daily dosing in children with HIV-associated tuberculosis: a pharmacokinetic and safety study within the open-label, multicentre, randomised, non-inferiority ODYSSEY trial
Source: Lancet HIV. 2022 Jul 19;9(9):e627–37. doi: 10.1016/S2352-3018(22)00160-6 (PMC9630157; doi:10.1016/S2352-3018(22)00160-6)
Supplement: Supplementary appendix [file mmc1.pdf]

THE LANCET
HIV

Supplementary appendix

This appendix formed part of the original submission and has been peer reviewed. We post it as supplied by the authors.

Supplement to: Turkova A, Waalewijn H, Chan MK, et al. Dolutegravir twice-daily dosing in children with HIV-associated tuberculosis: a pharmacokinetic and safety study within the open-label, multicentre, randomised, non-inferiority ODYSSEY trial. *Lancet HIV* 2022; published online July 19. https://doi.org/10.1016/S2352-3018(22)00160-6.

Table of Contents

ODYSSEY Trial Team 2

Table S1. Dolutegravir dosing in the ODYSSEY trial across different protocol versions 5

Table S2. Pharmacokinetic parameters of rifampicin of children with evaluable rifampicin pharmacokinetic curves by WHO paediatric TB weight band dosing 6

Table S3. Listing of adverse events reported 7

Table S4. Summary of adverse events, frequency and rates by dolutegravir dose 8

Figure S1. Timing of pharmacokinetic day visits and dolutegravir dosing in relation to tuberculosis treatment 9

Figure S2. Within-subject comparisons per age group and per dolutegravir dose for $AUC_{0\text{-}24h}$, C_{max} and C_{trough} for twice-daily dolutegravir with rifampicin and once-daily dolutegravir 10

Figure S3. Individual dolutegravir pharmacokinetic parameters on double dose dolutegravir co-administered with rifampicin by dolutegravir formulation and dose 11

ODYSSEY Trial Team

Penta Foundation: Carlo Giaquinto, Tiziana Grossele, Daniel Gomez-Pena, Davide Bilardi, Giulio Vecchia

Clinical Trial Units

MRC CTU at UCL: Shabinah S. Ali, Abdel Babiker, Shazia Begum, Chiara Borg, Anne-Marie Borges Da Silva, Joanna Calvert, Man Chan, Nimisha Dudakia, Deborah Ford, Joshua Gasa, Diana M. Gibb, Nasir Jamil, Sarah Lensen, Emma Little, Fatima Mohamed, Samuel Montero, Cecilia L. Moore, Rachel Oguntimehin, Anna Parker, Reena Patel, Tasmin Phillips, Tatiana Sarfati, Karen Scott, Clare Shakeshaft, Moira Spyer, Margaret Thomason, Anna Turkova, Rebecca Turner, Nadine Van Looy, Ellen White, Ian White, Kaja Widuch, Helen Wilkes, Ben Wynne

INSERM SC-10-US19--ANRS: Alexandra Compagnucci, Yacine Saidi, Yoann Riault, Alexandra Coelho, Laura Picault, Christelle Kouakam

PHPT: Tim R. Cressey, Suwalai Chalermpantmetagul, Dujrudee Chinwong, Gonzague Jourdain, Rukchanok Peongjakta, Praornsuda Sukrakanchana, Wasna Sirirungsi

Trial sites

Joint Clinical Research Centre, Uganda: Cissy M. Kityo, Victor Musiime, Elizabeth Kaudha, Annet Nanduudu, Emmanuel Mujyambere, Paul Ocitti Labeja, Charity Nankunda, Juliet Ategeka, Peter Erim, Collin Makanga, Esther Nambi, Abbas Lugemwa, Lorna Atwine, Edridah Keminyeto, Deogratiuos Tukwasibwe, Shafic Makumbi, Emily Ninsiima, Mercy Tukamushaba, Rogers Ankunda, Ian Natuhurira, Miriam Kasozi, Baker Rubinga, Diana Antonia Rutebarika, Rashidah Nazzinda, Shamim Nakabuye, Julius Tumusiime, Alice Mulindwa, Ritah Mbabazi, Milly Ndigendawani, Edward Bagirigomwa, Eddie Rubanga, David Eram, Maria Nannungi, Chrispus Katemba, Disan Mulima, Josephine Namusanje, Mariam Nabalamba, Priscilla Kyobutungi, Phyllis Mwesigwa Rubondo, Robinah Kibenge, Claire Nasaazi, Basiimwa Roy Clark, Enock Babu, Alex Musiime, Faith Mbasani, Martin Ojok, Odoch Denis, David Baliruno, Katabalwa Juliet, Benson Ouma, Barbara Ainebyoona, Mariam Naabalamba, Diana A. Rutebarika, Alex V. Musiime, Josephine Kobusungye, Ezra Lutalo

Baylor College of Medicine Children's Foundation, Uganda: Adeodata R. Kekitiinwa, Pauline Amuge, Dickson Bbuye, Justine Nalubwama, Winnie Akobye, Muzamil Nsibuka Kisekka, Anthony Kirabira, Gloria Ninsiima, Sylvia Namanda, Gerald M. Agaba, Immaculate Nagawa, Annet Nalugo, Florence Namuli, Rose J. Kadhuba, Rachael K. Namuddu, Lameck Kiyimba, Angella Baita, Eunice Atim, Olivia Kobusingye, Clementine Namajja, Africanus Byaruhanga, Rogers Besigye, Herbert Murungi, Geoffrey Onen, Lawrence Lekku, Judith Tikabibamu, Henry Balwa

MUJHU Research Collaboration, Uganda: Philippa Musoke, Linda Barlow-Mosha, Grace Ahimbisibwe, Rosemary Namwanje, Hajira Kataike, Mark Ssenyonga, Brenda Kakayi, Rebecca Sakwa, Sarah Nakabuye, Barbara Musoke Nakirya, Gladys Kasangaki, Raymonds Kyambadde, David Balamusani, Winnie Nansamba, Stella Nalusiba, Emmanuel Mayanja, Richard Isabirye, Erinah Kyomukama, Rebecca Wampamba, Mildred Kabasonga, Zaam Zinda Nakawungu, Sarah Babirye, Olivia Kaboggoza, Juliet Nanyonjo, Joanita Nankya Baddokwaya, Alice Elwana, Winfred Kaahwa, Bosco Kafufu, Emmanuel Hakiza, Maria Musisi, Paula Namayanja, Maria Gorreti Nakalema, Robert Serunjogi, Monica Etima, Phionah Kibalama, Joel Maena, Agnes Mary Mugagga, Annet Miwanda, Monica Nolan.

UZCRC, Zimbabwe: James Hakim, Hilda Mujuru, Kusum Nathoo, Mutsa Bwakura-Dangarembizi, Ennie Chidziva, Shepherd Mudzingwa, Secrecy Gondo, Godfrey Musoro, Vivian Mumbiro, Gloria Tinago, Shirley Mutsai, Joy Chimanzi, Columbus Moyo, Ruth Nhema, Misheck Nkalo Phiri, Stuart Chitongo, Joshua Choga, Joyline Bhiri, Wilber Ishemunyoro, Makhosonke Ndlovu, Moses Chitsamatanga, Pia Ngwaru, Tsitsi Gwenzi, Wendy

Mapfumo, Dorothy Murungu, Trust Mukanganiki, Prosper Dube, Tapiwa Gwaze, Farai Matimba, Tawona Mudzviti, Zivai Mupambireyi, Sibusisiwe Weza, Cleopatra Langa, Sandra Musarurwa, Shamiso Gwande

FAM-CRU, South Africa: Mark F. Cotton, Anita Janse van Rensburg, Marlize Smuts, Catherine Andrea, Sumaya Dadan, Sonja Pieterse, Vinesh Jeaven, Candice Makola, George Fourie, Kurt Smith, Els Dobbels, Peter Zuidewind, Hesti Van Huyssteen, Mornay Isaacs, Georgina Nentsa, Thabisa Ncgaba, Candice MacDonald, Maria Bester, Wilma Orange, Ronelle Arendze, Mark Mulder, Lucille Malgraaf, Ashley Harley, Yasmeen Akhalwaya

PHRU, South Africa: Avy Violari, Nastassja Ramsagar, Afaaf Liberty, Ruth Mathiba, Mandisa Nyati, Haseena Cassim, Lindiwe Maseko, Nkata Kekane, Busi Khumalo, Mirriam Khunene, Noshalaza Sbisi, Jackie Brown, Tryphina Madonsela, Nokuthula Mbadaliga, Zaakirah Essack, Reshma Lakha, Aasia Vadee, Derusha Frank, Nazim Akoojee, Maletsatsi Monametsi, Gladness Machache, Yolandie Fourie, Anusha Nanan-kanjee, Juan Erasmus, Angelous Mamiane, Tseleng Daniel, Fatima Mayat, Nomfundo Maduna, Patsy Baliram, Sibongile Sithebe, Emily Lebotsa, Siphiwe Mkhize

Klerksdorp Tshepong Hospital Complex, South Africa: Ebrahim Variava, Modiehi Rakgokong, Dihedile Scheppers, Tumelo Moloantoa, Abdul Hamid Kaka, Tshepiso Masienyane, Akshmi Ori, Kgosimang Mmolawa, Pattamukkil Abraham.

Durban International Clinical Research Site, South Africa: Moherndran Archary, Rosie Mngqibisa, Rejoice Mosia, Sajeeda Mawlana, Rashina Nundlal, Penelope Madlala, Allemah Naidoo, Sphiwee Cebekhulu, Petronelle Casey, Subashinie Sidhoo, Minenhle Chikowore, Lungile Nyantsa, Sheleika Singh

AHRI, South Africa: Nigel Klein, Osee Behuhuma, Olivier Koole, Kristien Bird, Nomzamo Buthelezi, Mumsy Mthethwa, Gugu Gasa, Siva Danaviah and Theresa Smit

PHPT CTU: Tim R. Cressey, Suwalai Chalermpantmetagul, Gonzague Jourdain, Nicole Ngo Giang Huong, Dujrudee Chinwong, Chalermpong Saenjum, Rukchanok Peongjakta, Pra-ornsuda Sukrakanchana, Woottichai Khamduang, Laddawan Laomanit, Ampika Kaewbundit, Jiraporn Khamkon, Kanchana Than-in-at, Sanuphong Chailert, Worathip Sripaoraya, Nitinart.krueduangkam, Namthip Kruenual, Warunee Khamjakkaew, Soraya Klinprung, **Prapokklao Hospital:** Chaiwat Ngampiyaskul, Pisut Greetanukroh, Praechadaporn Khannak, Pathanee Tearsansern, Wanna Chamjamrat, **Phayao Hospital, Thailand:** Nuttawut Chanto, Thitiwat Thapwai, Khanungnit Thungkham, Patcharee Puangmalai, Chutima Ruklao, **Chiangrai Prachanukroh Hospital, Thailand:** Pradthana Ounchanum, Suwimon Khusuwan, Sukanda Denjanta, Yupawan Thaweesombat, Jutarat Thewsoongnoen, Kanyanee Kaewmamueng, Phakamas Kamboua, Supawadee Pongprapass (Sangjan), Warunee Srisuk, Areerat Kongponoi, Juthamas Limplertjareanwanich, **Nakornping Hospital, Thailand:** Suparat Kanjanavanit, Prattana Leenasirimakul, Chayakorn Saewtrakool, Pacharaporn Yingyong, Duangrat Chutima (Suwan), Raungwit Junkaew, Orapin Khatngam, Thannapat Chankun, **Khon Kaen Hospital, Thailand:** Ussanee Srirompotong, Patamawadee Sudsaard, Sookpanee Wimonklang, Turian Petpranee, **Mahasarakam Hospital, Thailand:** Sathaporn Na-Rajsima, Pattira Runarassamee, Nuananong Kunjaroenrut, Arttasid Udomvised, Tassawan Khayanchoomnoom, Watchara Meethaisong, Ketmookda Trairat

HIVNAT, Thailand: Thanyawee Puthanakit, Suvaporn Anugulruengkitt, Wipaporn Natalie Songtaweesin, Torsak Bunupuradah, Naruporn Kasipong, Sararut Chanthaburanun, Apicha Mahanontharit, Kesdao Nanthapisal, Thidarat Jupimai, Thornthun Noppakaorattanamanee, Chutima Saisaengjan

European Site Investigators: Goethe University Frankfurt**, Germany:** Stephan Schultze-Strasser, Christoph Königs , **UKE Eppendorf, Germany:** Robin Kobbe, Ulf Schulze-Sturm, Felicia Mantkowski, Cornelius Rau, **Heartlands Hospital, UK:** Steve Welch, Jacqui Daglish, Laura Thrasyvoulou, Kate Gandhi, Yvonne Vaughan-Gordon, **Great Ormand Street Hospital, UK:** Delane Shingadia, Sophie Foxall, Judith Acero, Malgorzata Pasko-Szcech, Jacquie Flynn, **St Mary's Hospital, UK:** Gareth Tudor-Williams, Amina Farhana Mehar, Caroline Foster, Sobia Mustafa, **Leicester Royal Infirmary, UK:** Srini Bandi, Jin Li, Jackie Philps, **Leeds General Infirmary, UK:** Sean O'Riordan, Dominique Barker, Richard Vowden, Maria Dowie **Kings College Hospital, UK:** Colin Ball Eniola Nsirim, Kathleen McClaughlin, **Hospital 12 de Octubre, Spain:** India Garcia, Pablo Rojo Conejo, Cristina Epalza,

Luis Prieto Tato, Maite Fernandez, Luis Escosa Garcia, **Hospital La Paz, Spain:** Maria José Mellado Peña, Talia Sainz Costa, **Hospital San Joan de Déu, Spain:** Claudia Fortuny Guasch, Antoni Noguera Julian, Carolina Estepa, Elena Bruno, Patricia Mendez Garcia, Alba Murciano Cabeza, Biobanco Gregorio Maranon, Maria Angeles Muñoz Fernandez, Jose Luis Jimenez, Coral Gomez Rico, **Centro Materno-infantile do Norte, Portugal:** Laura Marques, Carla Teixeira, Alexandre Fernandes, Rosita Nunes, Helena Nascimento, Andreia Padrao, Joana Tuna, Helena Ramos, Ana Constança Mendes, Helena Pinheiro, Ana Cristina Matos

Local Site Monitors: Flavia Kyomuhendo, Sarah Nakalanzi, Cynthia Mukisa Williams, Leora Sewnarain, Ntombenhle Ngcobo, Deborah Pako, Nompumelelo Yende, Jacky Crisp, Marlize Smuts, Benedictor Dube, Precious Chandiwana, Winnie Gozhora, Thidarat Jumpimai

Substudies

PK substudies: David Burger, Pauline Bollen, Angela Colbers, Hylke Waalewijn, Tom Jacobs

Virology-immunology substudy: Nigel Klein, Eleni Nastouli, Anita De Rossi, Maria Angeles Munoz Fernandez, Carlota Miranda, Moira Spyer,

Social Science substudy and Youth Trial Board project: Janet Seeley, Sarah Bernays, Stella Namukwaya, Zivai Mupambireyi, Magda Conway, Lungile Jafta, Mercy Shibemba

Trial Committees

Independent Trial Steering Committee Members: Ian Weller, Elaine Abrams, Tsitsi Apollo, Polly Clayden, Valériane Leroy

Independent Data Monitoring Committee Members: Anton Pozniak, Jane Crawley, Rodolphe Thiébaut, Helen McIlleron

Endpoint Review Committee Members: Alasdair Bamford, Hermione Lyall, Andrew Prendergast, Felicity Fitzgerald, Anna Goodman

Table S1. Dolutegravir dosing in the ODYSSEY trial across different protocol versions

	ODYSSEY v2.0*	ODYSSEY v3.0		ODYSSEY v4.0		ODYSSEY v5.0 onwards	
	Main trial participants	Main trial participants	WB PK substudy participants	Main trial participants	WB PK substudy participants	Main trial participants	WB PK substudy participants
3-<6kg	-	-		-	5mg or 10mg DT[ψ]	-	5mg or 10mg DT[ψ]
6-<10kg	-	-		-	15mg DT	-	15mg DT
10-<14kg	-	-		-	20mg DT	-	20mg DT
14-<15kg	-	-	25mg FCT	25mg FCT→25mg DT[§]	25mg DT	25mg DT	N/A
15-<20kg	20mg FCT*	20mg FCT					
20-<25kg	25mg FCT	25mg FCT	25mg FCT	25mg FCT→30mg DT or 50mg FCT[¥]	30mg DT or 50mg FCT†	50mg FCT[π]	N/A
25-<30kg	25mg FCT	25mg FCT→50mg FCT**	50mg FCT	25mg FCT→50mg FCT**	NA	50mg FCT	NA
30-<35kg	35mg FCT	35mg FCT→50mg FCT**	50mg FCT	35mg FCT→50mg FCT**	NA	50mg FCT	NA
35-<40kg	35mg FCT	35mg FCT→50mg FCT**	50mg FCT	35mg FCT→50mg FCT**	NA	50mg FCT	NA
≥40kg	50mg FCT	50mg FCT	NA	50mg FCT	NA	50mg FCT	NA

DT=dispersible tablets, FCT=film-coated tables

* In May 2017 the EMA licensed the use of 20mg DTG in children 15 - <20kg and ≥6years, following this, children were able to be recruited in this weight and age-band.

** From 1st of April 2018, after ethics notification, sites following protocol version 3.0 and above were recommended to increase the DTG dose of children 25 - <40kg to 50mg FCT QD at their next scheduled study visit based on the results of the WB-PK2. WB-PK2 participants remained on DTG 50mg with ongoing follow-up. Non-PK participants recruited after implementation were initiated on the 50mg film-coated DTG dose.

ψ Infants <6 months of age received DTG 5mg QD while infants ≥6 months of age received DTG 10mg QD, both as dispersible tablets.

† Both doses are examined in WB-PK1 part II substudy in this weight-band.

§ Children 15 - <20kg previously receiving DTG 20mg QD were changed to DTG film-coated 25mg tablets upon the approval of protocol v4.0. Subsequently all children 14-<20kg changed to DTG 25mg QD dispersible tablets following the review of WB-PK1 part I results and approval by the relevant ethical and regulatory authorities.

¥ Children 20 - <25kg previously receiving DTG 25mg QD as one 25mg film-coated tablet changed to either DTG 30mg QD dispersible tablets or DTG 50mg QD film-coated tablet (depending on site) following the review of WB-PK1 part I results and approval by the relevant ethical and regulatory authorities.

π Following the review of PK and safety data children 20-<25kg receiving DTG 30mg dispersible tablets should be switched to DTG 50mg film-coated tablets. Those who prefer to remain on DTG 30mg DT will be able to do so until they move weight band.

Table S2. Pharmacokinetic parameters of rifampicin of children with evaluable rifampicin pharmacokinetic curves by WHO paediatric TB weight band dosing

WHO TB weight band	8-<12kg	12-<16kg	16-<25kg	≥25-<37kg†	≥37kg	All eligible children‡
N	1	1	4	8	4	18
Age at PK days, years	2.1	6.2	7.2 (6.9-8.9)	11.9 (10.4-13.1)	15.9 (15.2-16.0)	11.1 (7.5-14.5)
Weight, kg	9.5	14.6	20.1 (19.8-22.7)	31.3 (27.9-31.8)	45.6 (41.0-47.8)	29.7 (20.5-33.8)
RIF daily dose, mg/kg	15.8	15.4	14.9 (13.3-15.2)	10.4 (9.6-14.2)	11.6 (8.8-16.1)	13.8 (9.6-15.2)
GM C_{max} (CV%), mg/L*	6.5	12.9	3.3 (56)	4.7 (82)	7.0 (36)	5.1 (71)
AUC_{0-6}	19.3	36.0	9.1 (55)	14.0 (80)	26.8 (27)	15.9 (75)

Data are median (IQR) for age, weight, and rifampicin doses, and geometric means (coefficient of variation %) for pharmacokinetic parameters, unless indicated otherwise. $AUC_{0–24h}$=area under the concentration-time curve from 0 to 24 h. C_{max}=maximum plasma concentration. PK=pharmacokinetic. RIF=rifampicin. SEM= standard error for the mean.
*Rifampicin Cmax range: optimal 8-24 mg/L, low 4-<8 mg/L, very low <4mg/L (Peloquin et al., 2002). Of total 18 children with evaluable rifampicin concentrations, 3 children (17%) had optimal rifampicin Cmax, 11(61%) had low Cmax and 4(22%) very low Cmax.
†7 children included for AUC_{0-6} due to missed rifampicin sample at 6 hours after dose for one participant.
‡17 children included for AUC_{0-6} due to missed rifampicin sample at 6 hours after dose for one participant.

Reference: Peloquin CA. Therapeutic drug monitoring in the treatment of tuberculosis. Drugs. 2002;62(15):2169-83.

Table S3. Listing of adverse events reported

ID	TB-PK participation	Time from DTG BID to event (days)	DTG dose at event diagnosis date	SAE	System Level	Event Description	Event Grade	IRIS	ART Modifying	Relatedness to ART (ERC)
2	Done TB-PK	40	BID_35	Yes	Infectious Disease	Tuberculosis - disseminated/miliary	3	Yes	No	No
4	Done TB-PK	65	BID_25	No	Haematological	Neutropenia	3	N/A	No	No
5	Done TB-PK	184	QD_25¥	No	Infectious Disease	Hepatitis A	4	No	Yes#	No
6	Done TB-PK	105	BID_25	Yes	Infectious Disease	Acute febrile episode - undiagnosed	3	No	No	No
7	Done TB-PK	4	BID_25	Yes‡	Infectious Disease	URTI	2	N/A	No	No
7	Done TB-PK	4	BID_25	Yes‡	Skin	Rash, maculopapular	2	N/A	No	No
7	Done TB-PK	28	BID_25	No	Haematological	Anaemia with no clinical symptoms	3	No	No	No
8	Done TB-PK	16	BID_50	Yes	Nervous System	Epilepsy, fits, convulsions	3	No	No	No
8	Done TB-PK	28	BID_50	No	Haematological	Anaemia with clinical symptoms	3	No	No	No
8	Done TB-PK	28	BID_50	Yes	Cardiovascular	Deep vein thrombosis	3	No	No	No
8	Done TB-PK	126	BID_50	No	Infectious Disease	Tuberculosis - disseminated/miliary	3	Yes	No	No
1	Not done TB-PK	28	BID_20	Yes	Systemic	Kwashiorkor	4	Yes	No	No
1	Not done TB-PK	59	BID_20	Yes	Infectious Disease	Tuberculosis - disseminated/miliary*	5	Yes	No	No
3	Not done TB-PK	175	BID_50	No	Haematological	Neutropenia	3	No	No	No
9	Not done TB-PK	34	BID_35	Yes	Hepatic	Drug induced liver injury	4	No	No	No
10	Not done TB-PK	1	BID_50	Yes	Renal	Renal failure - chronic	4	No	No	No
11	Not done TB-PK	104	BID_10**	Yes	Non-HIV related death	Traumatic*	5	No	No	No

#DTG was stopped. This event was considered by the ERC to be unlikely/unrelated to DTG.
*SAE reported to have resulted in death.
‡Components of the same clinical SAE (Rash, maculopapular (grade 2) and URTI (grade 2))
¥Participant was returned to 25mg DTG once-daily 2 days before diagnosis of hepatitis A.
SAEs, serious adverse events; DTG, dolutegravir; BID, twice daily; ERC, endpoint review committee.
*All participants were receiving DTG film coated tablets at event diagnosis, except for **one child who was receiving 10mg dispersible tablets formulation.

Table S4. Summary of adverse events, frequency and rates by dolutegravir dose

ART regimen*	Safety follow-up** (person years)	SAEs		Grade 3 or above	
		N [N children]	Rate p.100PYs [95% CI]	N [N children]	Rate p.100PYs [95% CI]
**≥40Kg**					
Approved DTG dose	6.5	2 [1]	30.6 [3.7-110.7]	5 [2]	76.6 [24.9-178.8]
**<40Kg**					
Previously approved / Lower DTG dose#	10.1	6 [5]	59.6 [21.9-129.8]	8 [7]	79.5 [34.3-156.6]
Currently approved / Higher DTG dose##	8.4	2 [2]	23.8 [2.9-86.1]	2 [2]	23.8 [2.9-86.1]

* Weight-based DTG doses were given twice-daily, i.e., their daily dose was doubled when co-administered with rifampicin and until 2 weeks after rifampicin was stopped. Safety follow-up time is between starting twice-daily DTG and 30 days after returning to once-daily DTG or last follow-up visit if not returned to once-daily DTG. Two participants did not return to once-daily DTG due to death.
**Children contribute to follow-up whilst on a protocol-defined DTG dose in DTG arm. Follow-up and adverse events occurring whilst on non-per protocol doses or off these regimens do not contribute to this analysis (0.5 person years; no adverse events)
Lower DTG doses: the ODYSSEY trial opened with children on doses evaluated by the IMPAACT dose-finding study and/or approved by FDA and/or EMA (20mg film-coated tablets (FCTs) in weight-band 15<20kg, 25mg in 20-<30kg and 35mg in 30-<40kg).
Following ODYSSEY nested weight-band PK substudy results (Bollen et al., 2020; Waalewijn et al. 2022), children outside of the PK substudies were moved to higher DTG doses: 25mg DT in 14-<25kg, 50mg FCT in 20-<40kg. FDA and EMA dosing licenses were subsequently updated.

References:

Bollen PDJ, Moore CL, Mujuru HA, Makumbi S, Kekitiinwa AR, Kaudha E, et al. Simplified dolutegravir dosing for children with HIV weighing 20 kg or more: pharmacokinetic and safety substudies of the multicentre, randomised ODYSSEY trial. Lancet HIV. 2020;7(8):e533-e44.

Waalewijn H, Chan MK, Bollen PDJ, Mujuru HA, Makumbi S, Kekitiinwa AR, et al. Dolutegravir dosing for children with HIV weighing less than 20kg: pharmacokinetic and safety substudies nested in the multicentre, randomised ODYSSEY trial. Lancet HIV. 2022 Feb 18; S2352-3018(21)00292-7.

Figure S1. Timing of pharmacokinetic day visits and dolutegravir dosing in relation to tuberculosis treatment

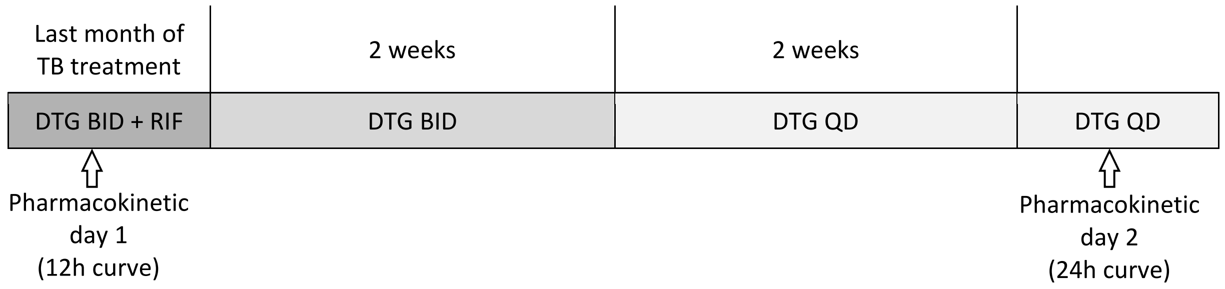

BID=twice daily. DTG=dolutegravir. QD=once daily. RIF=rifampicin. TB=tuberculosis

Figure S2. Within-subject comparisons per age group and per dolutegravir dose for AUC_{0-24h}, C_{max} and C_{trough} for twice-daily dolutegravir with rifampicin and once-daily dolutegravir

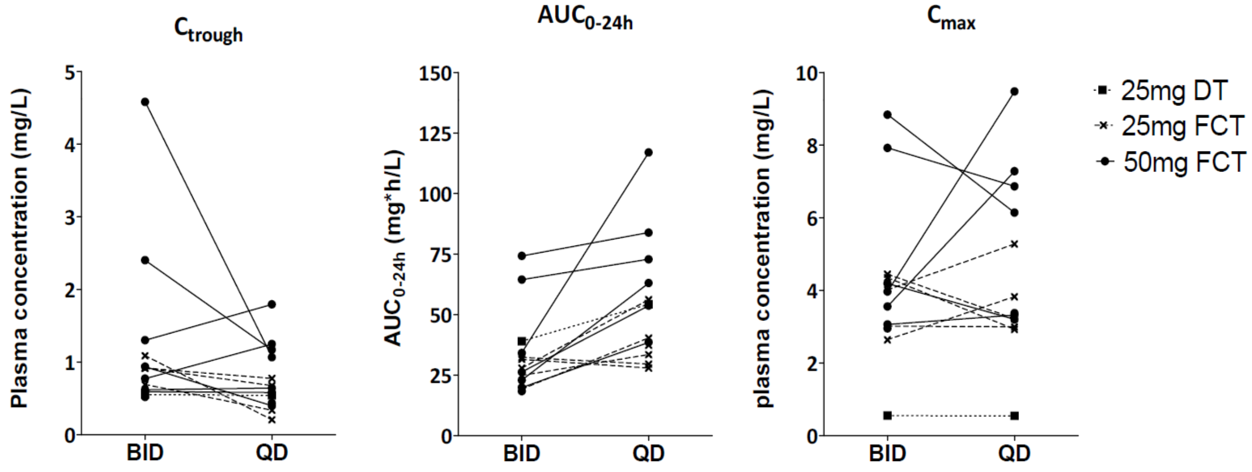

C_{trough}=concentration at the end of the dosing interval. AUC_{0-24h}=area under the concentration-time curve from dose until 24 hours after dose. C_{max}=highest measured concentration in dosing interval. BID=twice daily. QD=once daily.

Figure S3. Individual dolutegravir pharmacokinetic parameters on double dose dolutegravir co-administered with rifampicin by dolutegravir formulation and dose

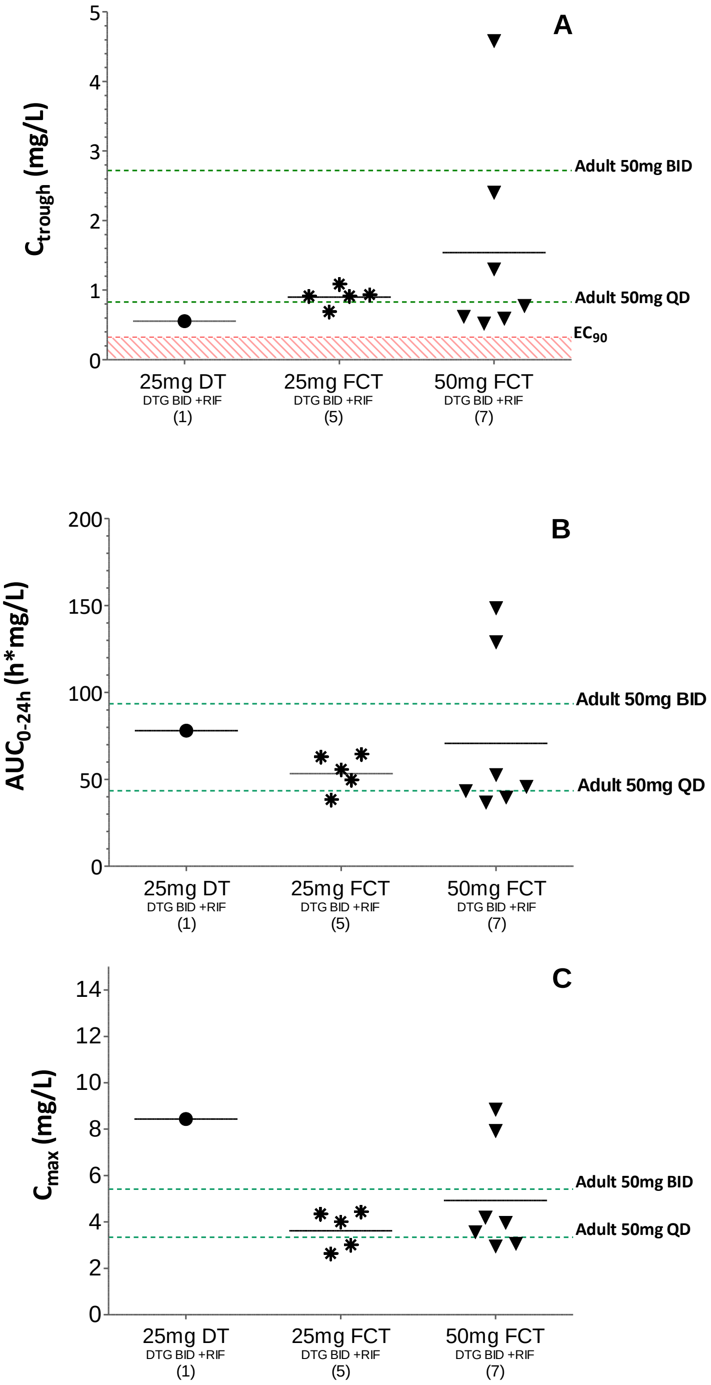

DT=dispersible tablets. FCT= film-coated tablets. C_{trough}=trough concentration. $AUC_{0–24h}$=area under the concentration-time curve from 0 to 24 h. C_{max}=maximum concentration. EC90= concentration at which 90% of maximal viral inhibition was achieved in a 10-day monotherapy study (Min et al., 2011). Individual dolutegravir C_{trough}, $AUC_{0–24h}$, and C_{max} in children on twice-daily dolutegravir taking 25mg DT, 25mg FCT or 50mg FCT co-administered with rifampicin. Horizontal black lines indicate geometric means per dose and formulation. Red dotted line indicates dolutegravir in-vivo EC90. Green dashed lines indicate geometric mean adult reference values for 50mg once-daily (lower line) and twice-daily (upper line) doses.

<u>Reference:</u> Min S, Sloan L, DeJesus E, Hawkins T, McCurdy L, Song I, et al. Antiviral activity, safety, and pharmacokinetics/pharmacodynamics of dolutegravir as 10-day monotherapy in HIV-1-infected adults. Aids. 2011;25(14):1737-45.
